# Representing Traditional East Asian Medicine in International Health Informatics Standards: A Scoping Review

**DOI:** 10.3390/healthcare14183011

**Published:** 2026-09-14

**Authors:** Seunggyeong Lee, Yeonju Woo, Soojin Lee

**Affiliations:** College of Korean Medicine, Sangji University, 83 Sangjidae-Gil, Wonju 26339, Republic of Korea; seung0322@sangji.ac.kr (S.L.); justice@sangji.ac.kr (Y.W.)

**Keywords:** traditional East Asian medicine, traditional medicine, interoperability, terminology, ICD-11, SNOMED CT, OMOP CDM, scoping review, health informatics

## Abstract

Background/Objectives: Traditional East Asian medicine (TEAM), comprising traditional Korean medicine (TKM), traditional Chinese medicine (TCM), and Japanese Kampo, is increasingly recorded in electronic health records, and sharing these data depends on interoperability standards. Yet, TEAM concepts rest on conceptual foundations differing from those of biomedical standards. Prior reviews examined traditional medicine’s own systems, not how TEAM concepts fit into mainstream biomedical standards. This review examined how TEAM concepts have been mapped or modeled into international health informatics standards. Methods: Following JBI methodology and PRISMA-ScR guidelines with a Population, Concept, and Context framework, we searched PubMed, Embase, Scopus, and IEEE Xplore for English reports from January 2016 to March 2026, with gray literature and citation searching. Screening was conducted by a single reviewer with senior verification and a 10% inter-rater reliability check. Findings were synthesized descriptively by functional category and clinical object type. Results: Of 16,763 records, 10 studies (2017 to 2026) were included; seven addressed TCM, three TKM, and none Kampo. Terminology work predominated, most often SNOMED CT, UMLS, and MeSH. The OMOP Common Data Model was the only data model, and no exchange standard such as HL7 FHIR appeared. Representability followed a gradient by clinical object type: acupuncture points anchored directly to terminologies, whereas only 1 of 260 ICD-11 Chapter 26 patterns had a SNOMED CT equivalent. Symptoms and disorders fit only partially, and compound herbal medicines required extension or decomposition. Most studies supplemented standards locally, and none of their additions were incorporated into a released standard. Conclusions: The work identified here has centered on establishing shared meaning rather than moving data between systems, and because supplementation stays external, the same gaps recur. Because the search covered only English-language, internationally indexed reports, these findings, including the absence of Kampo, reflect internationally visible work rather than the full literature published in Chinese, Korean, and Japanese national databases. Priorities are upstreaming TEAM content, encoding it in exchange standards, and building dedicated content for patterns.

## 1. Introduction

Traditional East Asian medicine (TEAM), which encompasses traditional Korean medicine (TKM), traditional Chinese medicine (TCM), and Japanese Kampo medicine (Kampo), is an established part of routine health care across East Asia [1]. TEAM care involves several distinct kinds of clinical object: interventions such as acupuncture points (acupoints) and compound herbal medicines; clinical findings such as symptoms; and diagnostic constructs such as traditional-medicine disorders and patterns, each a holistic classification of the patient’s overall systemic state [2]. Its place in mainstream practice was formally recognized in 2019, when the World Health Assembly adopted the eleventh revision of the International Classification of Diseases (ICD-11) with a dedicated chapter for traditional medicine, bringing TEAM concepts into a global health statistical classification for the first time [3]. TEAM clinical activity is increasingly recorded in electronic health records (EHRs) and research datasets and has become a subject of real-world data and artificial intelligence research [4,5]. For these data to support reproducible research, interdisciplinary collaboration, and integration with conventional care, they need to be represented in forms that can be shared and exchanged across systems rather than held in isolated repositories [6]. Representing TEAM data within these mainstream standards is therefore a precondition for sharing it across systems and using it alongside conventional care; the aim is to make the data interoperable, which complements rather than replaces traditional medicine’s own terminologies and frameworks.

Achieving this kind of shared, exchangeable representation depends on interoperability, the capacity of different information systems to exchange data and to use the information that has been exchanged [7]. In health care, interoperability standards can be organized into three functional categories: terminologies, classifications, and ontologies that establish shared clinical meaning; information or data models that structure clinical content for storage and analysis; and exchange standards that format and transmit data between systems [8]. Each of these is served by established standards. Clinical meaning is carried by terminologies and classifications such as Systematized Nomenclature of Medicine Clinical Terms (SNOMED CT) [9,10], the International Classification of Diseases 11th Revision (ICD-11) [2], the Unified Medical Language System (UMLS), Medical Subject Headings (MeSH), RxNorm (a standardized nomenclature for clinical drugs), and Logical Observation Identifiers Names and Codes (LOINC); structure is provided by common data models such as the Observational Medical Outcomes Partnership (OMOP) Common Data Model (CDM) [11]; and exchange is handled by messaging standards such as Health Level Seven Fast Healthcare Interoperability Resources (HL7 FHIR) [12,13]. Representing TEAM in an interoperable form therefore requires more than assigning a code to a concept; it involves expressing TEAM content coherently across all three categories.

Beyond expressing TEAM across these categories, a more fundamental difficulty arises: TEAM and biomedical standards rest on different conceptual foundations. Biomedical terminologies and classifications are built to represent well-defined clinical entities such as diseases, findings, and medications, whereas TEAM reasoning is organized around pattern differentiation, compound herbal medicines, and functional or relational constructs that do not correspond to single biomedical entities [14]. This conceptual distance adds to the difficulties that attend cross-terminology mapping even between biomedical vocabularies [15]. The ICD-11 traditional medicine chapter (Chapter 26, the Traditional Medicine Module I [TM1]) was itself a landmark, since traditional medicine had historically been excluded from the ICD [16]. Yet, this chapter represents only part of TEAM clinical practice [3], and the tension with the biomedical frame is visible in its design: its conditions are termed “disorders” rather than “diseases” because many TEAM entities are defined by clusters of signs and symptoms and not by a defined etiology [2,17]. How fully TEAM can be represented therefore varies with the type of clinical object at issue, from those that resemble biomedical entities to those that do not.

Prior reviews have approached the digitization of traditional medicine from several angles, but none has examined how TEAM clinical objects are represented in mainstream biomedical interoperability standards. Mirzaeian et al. [18] reviewed the progress and challenges of traditional medicine information systems, and Shojaee-Mend et al. [19] surveyed the methods used to develop and evaluate traditional medicine ontologies. More recently, Wang et al. [20] mapped the development of international standards and good practice guidelines specific to traditional, complementary, and integrative medicine, and Song et al. [21] described the state of TCM-specific terminology standards. These reviews share a focus on traditional medicine’s own systems, ontologies, and dedicated standards; that focus documents the internal digitization of traditional medicine but leaves open whether, and how, TEAM concepts can be carried by the mainstream standards that actually govern data exchange across health care. What remains unexamined is therefore how TEAM concepts fit into those mainstream biomedical standards, such as SNOMED CT, ICD-11, the OMOP CDM, and HL7 FHIR, and how that fit varies across the functional categories and the types of clinical object involved.

To address this gap, this scoping review examines how the concepts of traditional East Asian medicine have been mapped or modeled into international health informatics standards and with what methods, coverage, and outcomes. Its contribution beyond these prior reviews, which described traditional medicine’s own systems, is a cross-category map of TEAM representation in the mainstream standards: organizing a dispersed body of work by the functional categories engaged and the clinical object types addressed, it characterizes which TEAM clinical objects existing standards can represent, which they cannot, and how the field has responded where representation falls short.

## 2. Methods

### 2.1. Protocol and Registration

This scoping review followed the JBI methodology for scoping reviews [22] and is reported in accordance with the PRISMA extension for Scoping Reviews (PRISMA-ScR) [23] (File S1). It was further informed by the frameworks of Arksey and O’Malley [24] and Levac et al. [25]. The protocol was registered on the Open Science Framework (OSF) as an Open-Ended Registration on 25 May 2026 (registration ID: j2n75). The registration is currently under embargo and will be made publicly available, with a DOI assigned, upon publication. At the time of registration, the database searches, deduplication, and first-pass title and abstract screening had been completed, whereas the inter-rater reliability verification, the dual independent full-text screening, citation searching, and data charting were carried out after registration. Refinements made after registration, comprising additions to the data-charting form and changes to the planned analysis (the organization of outcomes by clinical object type, the axis of the coverage cross-tabulation, and the descriptive summary of validation approaches), are documented in a Registration Update on the same OSF project. This retrospective registration, and the subsequent update, are disclosed here and in the Limitations.

### 2.2. Eligibility Criteria

Eligibility was structured using the JBI Population, Concept, and Context (PCC) framework, adapted for a methodological scoping review of research artifacts. The population was data from TEAM, comprising the diagnostic and therapeutic paradigms of TKM, TCM, and Kampo, corresponding to the scope of WHO ICD-11 Chapter 26, Module I. The concept was mapping or modeling work whose primary contribution was the representation of TEAM data within or across international health informatics standards. The context was the health informatics and international standardization environment in which TEAM data are integrated into global health terminology and implementation standards. The full inclusion and exclusion criteria are presented in Box 1.

Box 1 **Inclusion and exclusion criteria**

Inclusion criteria

▯ Addressed the traditional East Asian medicine (TEAM) paradigms defined above, regardless of the location of the institution conducting the work.
▯ Mapped, modeled, developed an ontology for, or integrated TEAM data into international health content and implementation standards (for example, ICD-10 or ICD-11, the OMOP CDM, SNOMED CT, RxNorm, or HL7 FHIR), where this was the primary contribution and an explicit deliverable, such as a mapping table, mapping rules, an integration model, or an ontology artifact, was presented.
▯ Were peer-reviewed original articles or full papers in the regular proceedings of major medical informatics or biomedical venues, such as IEEE, AMIA, or MEDINFO.
▯ Were published between 1 January 2016 and 31 March 2026.
▯ Were written in English.

Exclusion criteria

▯ Addressed other traditional medicine systems outside the scope of ICD-11 Chapter 26, such as Ayurveda, Unani, or Siddha.
▯ Did not present mapping, modeling, or integration as the primary contribution, including simple comparative analyses, system implementation reports without a mapping methodology, standard-conformance work, and incidental mentions of a standard.
▯ Had as their primary work the alignment of TEAM concepts with an upper or foundational ontology, such as the Basic Formal Ontology or the General Formal Ontology.
▯ Were conference abstracts without a full paper, protocols, editorials, letters, or news items, or commentaries or perspectives without a methods section.
▯ Addressed a broad complementary and integrative health category in which results specific to TEAM data were not reported separately.

The eligibility criteria were operationalized and refined during first-pass title and abstract screening, consistent with the iterative nature of scoping review methodology [22,25], and were finalized before the inter-rater reliability verification. The English-language restriction reflected the review team’s language capacity and the focus on work disseminated to the international informatics community, and its implications are addressed in the Limitations.

### 2.3. Information Sources

Four bibliographic databases were searched: PubMed, Embase, Scopus, and IEEE Xplore. IEEE Xplore was included to capture medical informatics conference proceedings. The Cochrane Library was considered but not used as a source because it returned no native records of an eligible type, and the 223 records retrieved were trial records duplicated from the other databases. The searches were conducted in April 2026 and were limited to records published between 1 January 2016 and 31 March 2026. This window covers the most recent decade, during which the international data-model and exchange standards relevant to this review became established alongside the ICD-11 Chapter 26. In a confirmatory check performed during revision, we re-ran the PubMed strategy with the lower date limit removed (records dated 2015 or earlier, *n* = 1553) and screened the results against the same eligibility criteria; no eligible study was identified. For PubMed and Embase, the date limit was applied as an exact range; for Scopus and IEEE Xplore, it was applied at the year level, and records outside the range were removed during screening under the label Out of Timeframe.

A structured gray literature search was also carried out in May 2026 to confirm that coverage of governance bodies and institutional outputs was complete. Three categories of source were searched: international standards and governance bodies (for example, WHO IRIS, ISO Technical Committee 249, OHDSI, SNOMED International, HL7, and the National Library of Medicine), national and regional institutional repositories (for example, the Korea Institute of Oriental Medicine (KIOM), the National Institute for Korean Medicine Development, and the China Academy of Chinese Medical Sciences), and dissertation databases (ProQuest Dissertations and Theses Global). A small number of governance and institutional outputs were retained as background for the Discussion.

In addition to database searching, both backward and forward citation searching was performed on each study included after full-text screening to identify further eligible reports.

### 2.4. Search Strategy

The search strategy combined two concept blocks with the Boolean operator AND: terms for Traditional East Asian Medicine and terms for health informatics standardization and interoperability. The standardization block was built around broad concepts, such as interoperability, terminology, controlled vocabulary, ontology, data model, and mapping, together with named standards that were included to increase sensitivity. The full database-specific search strategies, including all search terms, field tags, and the date each database was searched, are provided in File S2. Standards not named in the search, such as UMLS, RxNorm, LOINC, and MeSH, were captured through the general concept terms; conversely, terms included only to increase sensitivity, such as ISO standards and the International Organization for Standardization, did not by themselves confer eligibility. Work whose primary contribution was the development of a traditional-medicine standard in its own right, including ISO Technical Committee 249 deliverables, fell outside this review’s concept, which concerned the mapping or modeling of TEAM data into mainstream international health informatics standards; accordingly, standard documents or conformance work without a mapping deliverable were not eligible. Consistent with this, the gray literature search of ISO Technical Committee 249 returned no records meeting the eligibility criteria.

### 2.5. Selection of Sources of Evidence

Records were imported into Rayyan [26] and deduplicated by algorithmic detection followed by manual checking. Before formal screening began, the primary reviewer and the senior reviewer screened a small set of records together to calibrate their application of the eligibility criteria and the hierarchical labeling scheme; this calibration exercise led to minor refinements of the label definitions before full screening started. Title and abstract screening was then conducted by the primary reviewer. To avoid contaminating the subsequent blinded reliability check, case-by-case consultation with the senior reviewer was not used during screening; where eligibility could not be determined from the title and abstract, a record was conservatively labeled Maybe rather than excluded and was carried forward to full-text assessment. At the title and abstract stage, excluded records were assigned a label in the following priority order: Wrong Language, Out of Timeframe, Wrong Publication Type, and Out of Scope. Full-text assessment was performed independently by 2 reviewers, and disagreements were resolved by consensus. Reports identified through citation searching were screened at full text independently by the same 2 reviewers against the same criteria.

This selection model represents a deliberate, resource-based deviation from the JBI recommendation of two or more independent reviewers at both stages [22]; PRISMA-ScR recognizes a single reviewer with a second verifier as an alternative to fully duplicate screening [23]. The deviation was compensated for by the retrospective inter-rater reliability verification described below and by dual independent full-text screening. The conservative carry-forward of uncertain records as Maybe was intended to reduce the risk of false-negative exclusions.

### 2.6. Inter-Rater Reliability Verification

After registration, inter-rater reliability for title and abstract screening was verified on a single random 10% sample of the full set of screened records, not stratified by screening category, generated with the Rayyan data-sampling function. Records used in the calibration exercise were excluded from this sampling frame, so no previously discussed record could inflate the observed agreement. The senior reviewer re-screened the sample independently in Rayyan blind mode, and agreement was computed outside Rayyan across the three screening categories. Because the proportion of included records was very low, Cohen’s kappa can be downwardly biased under skewed marginal distributions [27]. We therefore report the raw percent agreement and the number and direction of disagreements alongside Cohen’s kappa, together with the prevalence-adjusted bias-adjusted kappa (PABAK) [28] and Gwet’s AC1 [29], and we base the assessment of agreement primarily on the percent agreement and on the number of false-negative exclusions recovered rather than on kappa alone. Where the Landis and Koch [30] interpretive bands are referenced, they are read with this prevalence caveat in mind [31,32]. The paired screening decisions were compiled in Excel, and the contingency table and the agreement statistics (raw percent agreement, Cohen’s kappa, PABAK, and Gwet’s AC1) were computed in Python (version 3.13). The protocol specified reporting Cohen’s kappa with a 95% confidence interval; because the sample contained a single disagreement under an extreme prevalence imbalance, a confidence interval for kappa was not estimable under the large-sample normal approximation, and the coefficients are reported as point estimates. The full agreement contingency table and the corresponding computations are provided in Table 1 and Table S1.

### 2.7. Data Charting and Data Items

A standardized data charting form was used, following the JBI 2024 [22] recommended extraction items and adapted to the review topic. Charting was performed by the primary reviewer, and the charted data were verified against the source reports by the senior reviewer, consistent with the single-reviewer-with-verification model supported by JBI 2024 [22]. In line with JBI guidance, charting was treated as an iterative process: the form was piloted on a small set of included records and refined as the review proceeded. The data charting form is provided in Table S2.

The registered form captured twelve fields: record identifier; author and year; country; publication venue; tradition; study type; source data; target standard or standards; mapping or modeling methodology; primary contribution; key quantitative outcomes; and whether code or data were available in an open repository. Three further fields were added during charting: standardization level, disease or domain scope, and tools, software, or platforms. Consistent with the iterative charting expected in scoping reviews, these additions are documented in the Registration Update described above.

Two charted variables were defined operationally. The standardization level classified each study as terminology-level or information-model-level work, following the terminology and information-model distinction in health informatics interoperability [9]. The level of automation of the mapping or modeling process was classified as manual, semi-automatic, or automatic according to the degree of human involvement [15,33].

Although the protocol specified the online publication date for determining publication year, the journal volume year was used for 1 study ([34]; online 2018, volume 2019) to align with citation and indexing conventions. This affected only the temporal placement of a single study and no eligibility decision.

### 2.8. Critical Appraisal

Consistent with scoping review methodology, the methodological quality and risk of bias of the included sources were not appraised, because the aim was to map the extent and nature of existing work and to identify gaps rather than to assess quality [22,23]. For the same reason, neither the publication format nor the journal indexing status of a source was used as an eligibility or quality criterion. The included sources span journal articles and peer-reviewed conference proceedings, and because the search used four databases together with citation searching, eligibility did not depend on indexing in any single database; full citation details for all ten studies are provided in Table 2 and the reference list so that each can be located.

### 2.9. Synthesis of Results

The charted data were synthesized descriptively, following JBI 2024 [22]; no formal, meta-analytic, or thematic synthesis was undertaken. Frequency analyses were performed by publication year, country, tradition, target standard, and automation level; validation approaches were summarized descriptively rather than counted, given their heterogeneity. The standards addressed by each study were organized into three functional categories, namely terminology or semantic, data model or content, and exchange or transport, consistent with the ONC ISA, and were summarized in a coverage matrix that cross-tabulated studies by standard and by tradition. In addition, the studies’ reported mapping outcomes were organized by the type of clinical object represented (acupoints, symptoms, disorders, compound herbal medicines, and pattern) to characterize how representability varied across object types; because the studies used heterogeneous and not directly comparable metrics, this dimension was summarized descriptively rather than pooled. This organization was introduced during analysis and, like the charting-form refinements, is documented in the Registration Update. The distribution of studies by publication year and tradition was also summarized graphically.

## 3. Results

### 3.1. Selection of Studies

The database search identified 16,763 records (PubMed, *n* = 3115; Embase, *n* = 9308; Scopus, *n* = 3955; IEEE Xplore, *n* = 385). After 5862 duplicates were removed, 10,901 records remained for title and abstract screening, of which 10,878 were excluded, leaving 23 reports (15 included and 8 marked as uncertain) for full-text assessment. Citation searching (snowballing) identified a further 239 records, of which 234 were excluded at title and abstract screening, leaving 5 reports for full-text assessment. All 28 reports sought for retrieval were obtained. The gray literature search identified no additional records meeting the eligibility criteria.

**Figure 1:**
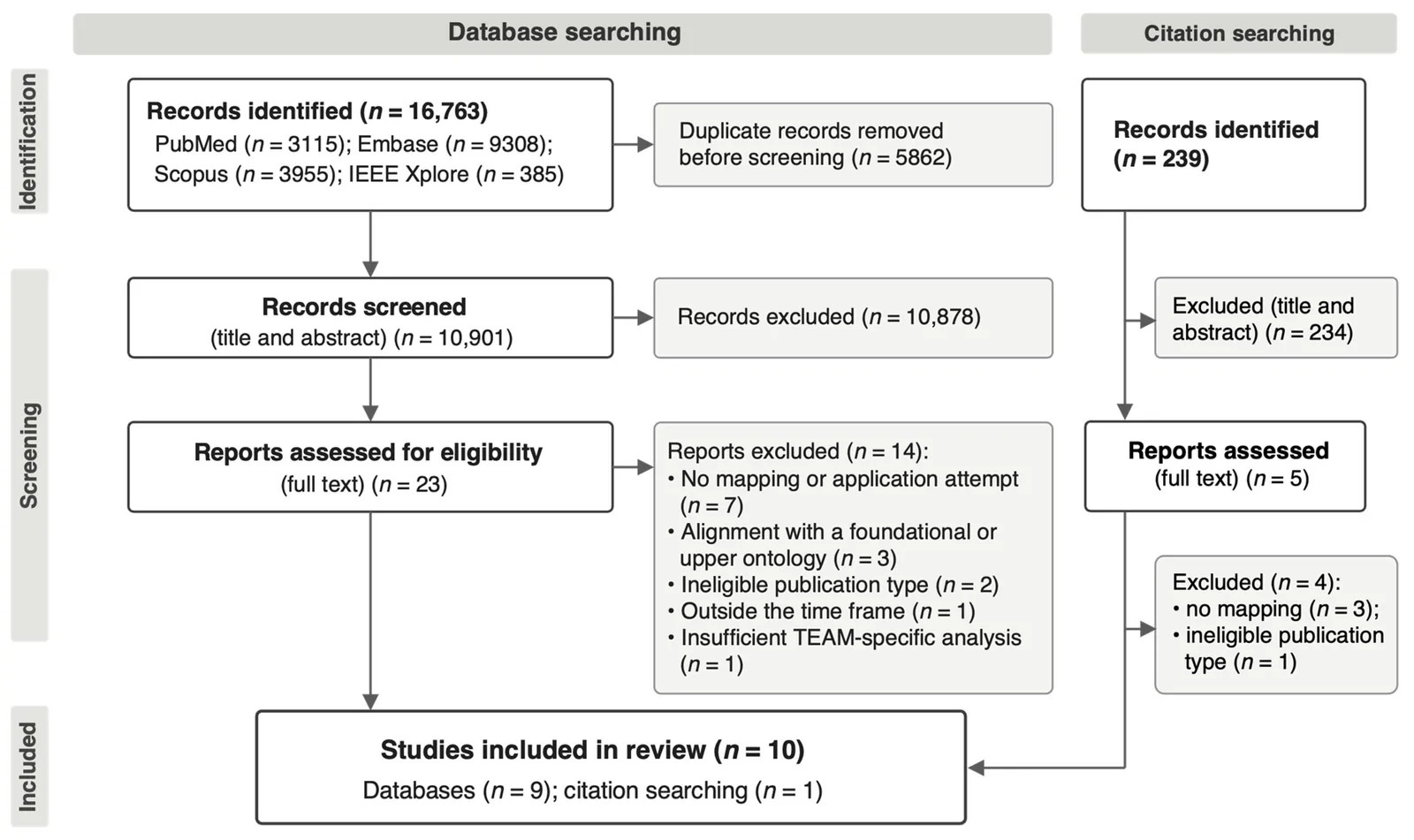
PRISMA-ScR flow diagram of the study selection process. Studies were identified through systematic searching of four bibliographic databases (PubMed, Embase, Scopus, and IEEE Xplore) and through backward and forward citation searching of the included studies. Reports excluded at the full-text stage are listed with reasons. TEAM, traditional East Asian medicine.

At the full-text stage, 14 of the 23 database-derived reports were excluded (no mapping or application attempt, *n* = 7; alignment with a foundational or upper ontology, *n* = 3; ineligible publication type, *n* = 2; outside the time frame, *n* = 1; insufficient TEAM-specific analysis, *n* = 1), and 4 of the 5 citation-derived reports were excluded (no mapping or application attempt, *n* = 3; ineligible publication type, *n* = 1). Ten studies met the eligibility criteria: 9 from the database search and 1 (Byun et al. [35]) identified through forward citation tracking of Lundholm et al. [36]. The selection process is summarized in Figure 1. A list of the reports excluded at full-text assessment, with the primary reason for each, is provided in Table S3.

Inter-rater reliability for title and abstract screening was verified retrospectively on a single random 10% sample of the 10,901 screened records (*n* = 1090), not stratified by screening category, which the senior reviewer re-screened independently in Rayyan under blind mode. Because the sample was drawn from the full set, its composition reflected the underlying distribution, in which excluded records predominated (1087 of 1090). The reviewers agreed on 1089 of the 1090 records (raw agreement, 99.91%); the single disagreement concerned a record that one reviewer marked for inclusion and the other marked as uncertain, and both categories were carried forward to full-text assessment, so no eligible record was excluded at this stage. The full agreement contingency table is shown in Table 1. Because included records were very rare in the sample (3/1090, 0.28%), Cohen’s kappa is downwardly biased under the skewed marginal distribution, and prevalence-adjusted measures were reported alongside it; its confidence interval was not estimable under this prevalence imbalance. The agreement statistics were high but were dominated by the large majority of concordant exclusions (Cohen’s kappa = 0.833; PABAK = 0.998; Gwet’s AC1 = 0.999).

**Table 1:** Inter-rater reliability of title and abstract screening on a 10% random verification sample (*n* = 1090). R1 = primary reviewer, R2 = senior verifying reviewer.

	R2: Include	R2: Maybe	R2: Exclude	Row Total
R1: Include	2	1	0	3
R1: Maybe	0	0	0	0
R1: Exclude	0	0	1087	1087
Column total	2	1	1087	1090

Note. The single disagreement concerned one record marked Include by R1 and Maybe by R2; both categories were carried forward to full-text assessment, so no eligible record was excluded at this stage. Full computations are provided in Table S1.

### 3.2. Characteristics of Included Studies

**Figure 2:**
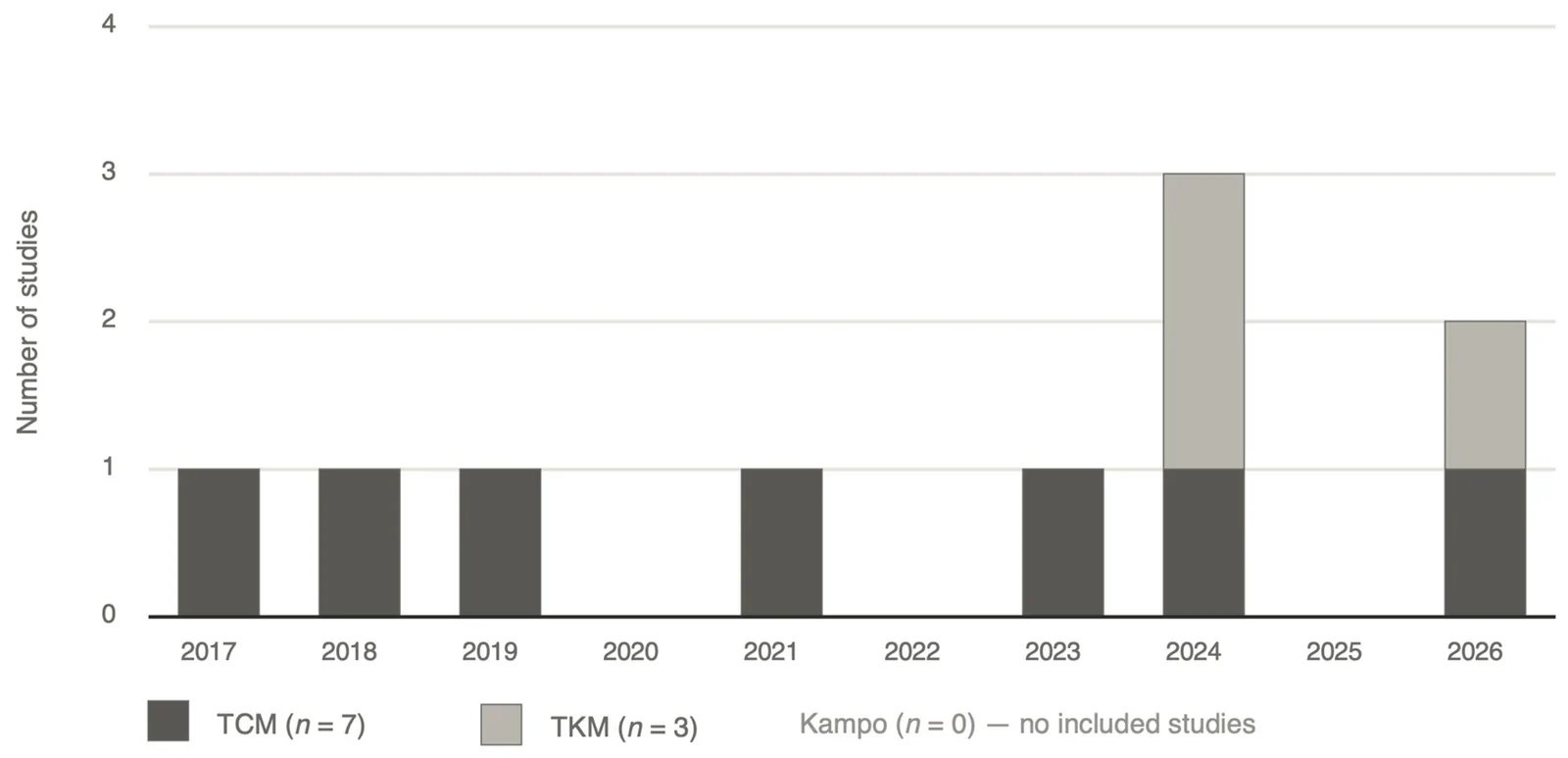
Distribution of the included studies by publication year and medical tradition (*n* = 10). Bars are stacked by medical tradition (traditional Chinese medicine and traditional Korean medicine). No included study addressed Kampo. Note. Publication year reflects the journal volume of record. Wu et al. (2019) [34] appeared online in 2018 but was assigned to the 2019 volume; the volume year is used here for consistency with the reference list.

The ten included studies were published between 2017 and 2026; they appeared across the period rather than in a single burst, with the largest number in 2024 (*n* = 3) and 2 in 2026 (Figure 2). They were conducted in China (*n* = 3), Republic of Korea (*n* = 3), and Sweden (*n* = 1), together with 3 multi-national collaborations. A total of 7 studies addressed TCM, and 3 addressed TKM; no study addressed Kampo. The studies drew on heterogeneous source data, including clinical practice guidelines, electronic medical records (EMRs), patient registries, classical texts, and drug databases, and they ranged from feasibility demonstrations and data-model conversions to terminology mapping, ontology or knowledge-graph construction, and database integration. These sources differed markedly in scale, from small, curated corpora of guidelines or classical texts to a full institutional electronic health record of 88,449 patients [37], the largest patient-level dataset among the included studies. A total of 5 of the 10 studies deposited their outputs in a public repository [34,36,38,39,40], and 1 further study [37] released its conversion code while restricting the underlying patient data and the institutional terminology; the remaining 4 did not provide openly accessible outputs. Full characteristics of the included studies are presented in Table 2.

**Table 2:** Characteristics of the included studies (*n* = 10).

No.	Study (Author, Year)	Country	Medical Tradition	Source Data	Target Standard(s)	Primary Contribution	Open Repository
1	Cha J (2024) [41]	Republic of Korea	TKM	Clinical practice guidelines	OMOP CDM	Feasibility of OMOP CDM for KM guideline data	No
2	Chang A (2021) [42]	Sweden, China	TCM	Patient registry	ICD-11	Dual coding of low back pain across ICD-11 CM and TM1 sections	No
3	Li Y (2026) [38]	USA, China	TCM	Web resources, literature, ontologies	SNOMED CT, MeSH	AcuKG linked to biomedical ontologies	Yes
4	Lundholm T (2023) [36]	Sweden	TCM	ICD-11 TM1 concepts	SNOMED CT	Evaluates ICD-11 TM1 representability in SNOMED CT; builds a TM ontology	Yes
5	Park MY (2026) [37]	Republic of Korea	TKM	Korean medicine hospital EHR	OMOP CDM; SNOMED CT, RxNorm, LOINC	Full KM-hospital EHR to OMOP CDM, with a reusable TKM terminology extension	Partial ^a^
6	Ruan T (2017) [39]	China	TCM	Web encyclopedia websites, hospital EMR	UMLS	Automated Chinese symptom knowledge base linked to UMLS	Yes
7	Shu Z (2024) [40]	China	TCM	Inpatient EMRs, classical texts, vocabularies	UMLS, MeSH, ICD-11	ISPO bridging TCM and biomedical terms	Yes
8	Wang L (2018) [43]	China, USA	TCM	Chinese drug registries, pharmacopoeia	RxNorm	NCCD: RxNorm extension for Chinese patent drugs	No ^b^
9	Wu Y (2019) [34]	China	TCM	Pharmacopoeia	UMLS, MeSH	SymMap: TCM symptoms mapped to UMLS, with phenotype/molecular links	Yes
10	Byun A (2024) [35]	Republic of Korea	TKM	Clinical practice guidelines	SNOMED CT	TKM migraine concepts to SNOMED CT; proposes SNOMED extensions	No

^a^ Park MY (2026) [37]: ETL code publicly available on GitHub (https://github.com/ManYoungPark/KCDM_OMOP); patient data and full KIOM terminology not released; representative mapping rules provided in the original study’s Supplementary Materials. ^b^ Wang L (2018) [43]: link inactive. Note. The complete charting record, together with the mapping methodology, validation, tools, and reported quantitative outcomes, is provided in Table S4, and the methodology and validation are synthesized in Table 3. The Target Standard column lists only the international health informatics standards addressed by each study; auxiliary ontologies and vocabularies used during ontology, knowledge-graph, or database construction are listed in Table S5. Abbreviations: AcuKG, Acupuncture Knowledge Graph; CM, conventional medicine; EHR, electronic health record; EMR, electronic medical record; ICD-11, International Classification of Diseases 11th Revision; ISPO, Integrated Symptom Phenotype Ontology; KM, Korean medicine; LOINC, Logical Observation Identifiers Names and Codes; MeSH, Medical Subject Headings; NCCD, Normalized Chinese Clinical Drug knowledge base; OMOP CDM, Observational Medical Outcomes Partnership Common Data Model; RxNorm, standardized nomenclature for clinical drugs (U.S. National Library of Medicine); SNOMED CT, Systematized Nomenclature of Medicine Clinical Terms; SymMap, Symptom Mapping database; TCM, traditional Chinese medicine; TKM, traditional Korean medicine; TM, traditional medicine; TM1, Traditional Medicine Module I; UMLS, Unified Medical Language System.

### 3.3. Coverage of International Standards

**Figure 3:**
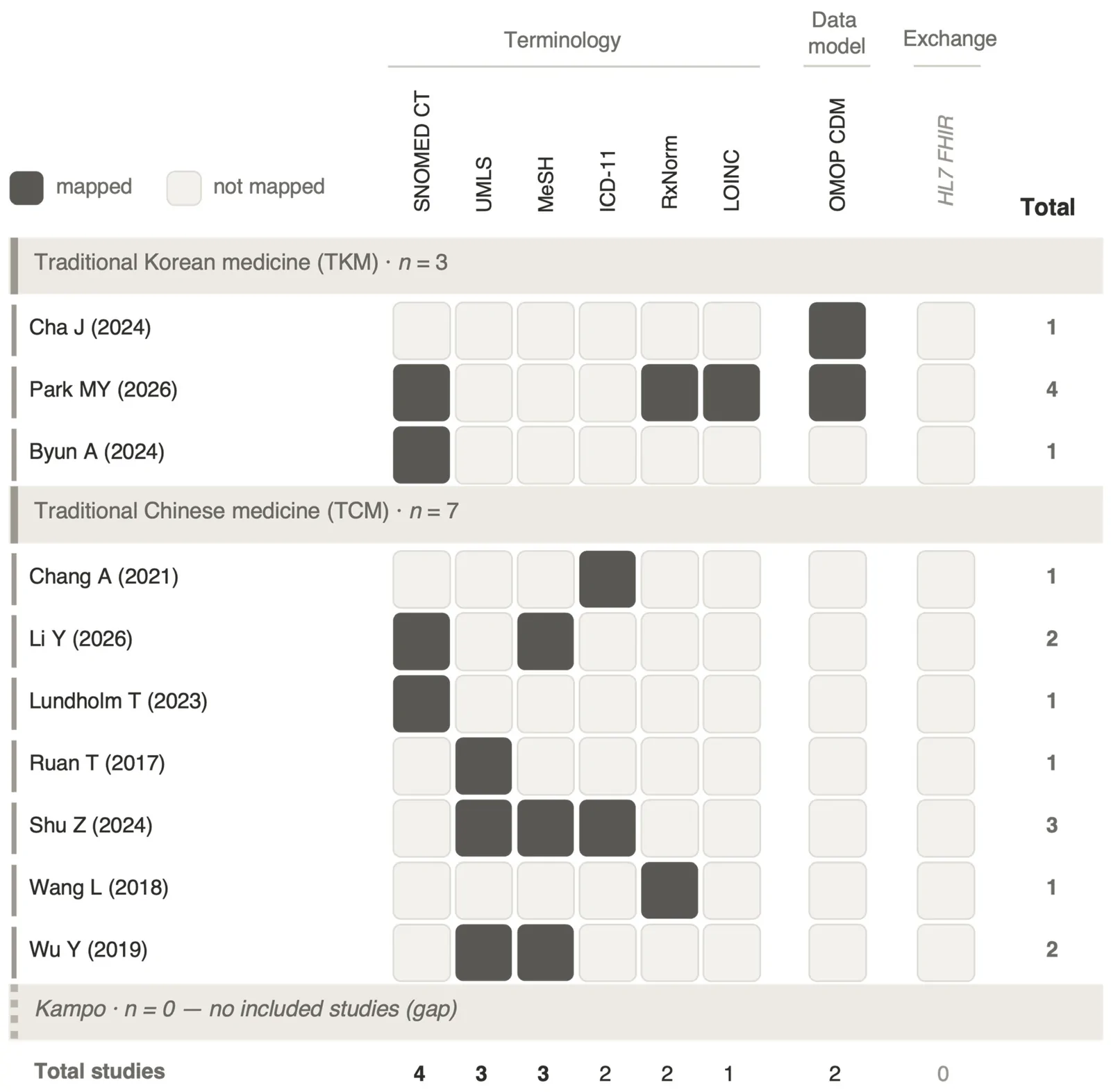
Coverage matrix of mappings between traditional East Asian medicine (TEAM) data and international health informatics standards across the included studies (*n* = 10). Columns are grouped by interoperability category (terminology, data model, and exchange). Rows are the included studies grouped by tradition. Filled cells (dark grey) indicate a reported mapping; empty cells (light grey), including the exchange column (for which HL7 FHIR is the most prominent standard) and the Kampo row, denote standards or traditions within the review’s scope for which no included study reported a mapping. The Total column reports the number of standards mapped by each study, and the Total studies row reports the number of studies that mapped to each standard. The 10 included studies shown as row labels are: Byun A (2024) [35], Cha J (2024) [41], Chang A (2021) [42], Li Y (2026) [38], Lundholm T (2023) [36], Park MY (2026) [37], Ruan T (2017) [39], Shu Z (2024) [40], Wang L (2018) [43], Wu Y (2019) [34].

Across the 10 studies, mapping and modeling efforts were concentrated in the terminology category, were sparse in the data-model category, and were absent from the exchange category (Figure 3). SNOMED CT was the most frequent target (*n* = 4), followed by UMLS (*n* = 3) and MeSH (*n* = 3); ICD-11 (*n* = 2) and RxNorm (*n* = 2) were each addressed twice, and LOINC once. In the data-model category, the OMOP CDM was the only common data model engaged, appearing in 2 studies. No included study engaged an exchange or messaging standard such as HL7 FHIR, so this category remained unrepresented.

Most studies engaged a single standard; the studies that engaged several [34,37,38,40] corresponded to data-model conversion, ontology and knowledge-graph construction, and database integration. Beyond the international standards shown in Figure 3, several studies also drew on ontologies and vocabularies (for example, UBERON, the Symptom Ontology, the Human Phenotype Ontology, SIDER, OMIM, and Orphanet) as auxiliary resources during ontology, knowledge-graph, or database construction; these are not international health informatics interoperability standards and are not represented in the coverage matrix. Two gaps were apparent: no study addressed the exchange category (whose most prominent standard is HL7 FHIR), and none represented the Kampo tradition.

### 3.4. Mapping and Modeling Methodology and Validation

The standardization level achieved varied across studies (Table 3). Eight studies operated at the terminology level, binding TEAM concepts to reference terminologies, vocabularies, or ontologies; 1 study [41] operated at the information-model level by fitting TEAM data to the OMOP CDM; and 1 study [37] addressed both the terminology and information-model levels.

The mappings were produced largely by hand. A total of 5 studies used manual mapping, 4 used semi-automatic approaches that combined algorithmic candidate generation with expert review, and 1 study [39] a predominantly automatic pipeline. Validation practices were heterogeneous and not directly comparable, and the reported outcomes likewise reflect different reference points rather than a shared benchmark. Reported approaches ranged from feasibility assessment without a formal metric [41] and qualitative case demonstration [42] to coverage assessment [36,40], accuracy or precision metrics [39,43], downstream-task accuracy (the accuracy of a knowledge-graph-augmented question-answering task) [38], and expert-consensus curation [34]. One study [35] reported a reviewer-agreement rate (percent agreement). The methodological and validation characteristics of each study are detailed in Table 3.

**Table 3:** Methodological characteristics and validation of the mapping and modeling approaches in the included studies (*n* = 10).

No.	Study (Author, Year)	Standardization Level	Representation Form	Automation Level	Primary Validation Type
1	Cha J (2024) [41]	Information model	OMOP CDM transformation	Manual	Feasibility (no metric)
2	Chang A (2021) [42]	Terminology	Terminology coding (ICD-11)	Manual	Qualitative expert assessment
3	Li Y (2026) [38]	Terminology	Knowledge graph	Semi-automatic	Downstream-task accuracy
4	Lundholm T (2023) [36]	Terminology	Ontology (OWL)	Manual	Coverage assessment
5	Park MY (2026) [37]	Terminology and information model	OMOP CDM + terminology extension	Semi-automatic	Coverage + data quality
6	Ruan T (2017) [39]	Terminology	Knowledge base	Automatic ^a^	Accuracy + precision
7	Shu Z (2024) [40]	Terminology	Ontology (OWL/RDF)	Semi-automatic	Coverage + expert consensus
8	Wang L (2018) [43]	Terminology ^b^	Knowledge base (RxNorm model extension)	Semi-automatic	Accuracy + coverage
9	Wu Y (2019) [34]	Terminology	Integrative database	Manual ^c^	Expert consensus
10	Byun A (2024) [35]	Terminology	Terminology mapping	Manual	Reviewer agreement ^d^

^a^ Ruan T (2017) [39]: classified as Automatic because entity extraction and the linking of the knowledge base to the UMLS are algorithm-driven and the study self-describes as an “automatic approach”; data schema design and training annotation were manual, so the classification is sensitive to the axis chosen (mapping engine versus training involvement). ^b^ Wang L (2018) [43]: classified as Terminology-level because the core deliverable is drug normalization to RxNorm; the study additionally extended the RxNorm information model with a new patent-name semantic type (PN), which could alternatively support classification as both terminology-level and information-model-level. ^c^ Wu Y (2019) [34]: the mapping of TCM symptoms to the UMLS was manually curated by a committee of 17 experts (≥2-of-3 consensus per term); the statistical inference (Fisher exact test with false discovery rate control) applies to indirect symptom–symptom and molecular associations, not to the symptom–standard mapping itself. ^d^ Byun A (2024) [35]: the reported values are the percent agreement between the mapper and the reviewer, not a kappa-based inter-rater statistic, and external validation covered a 26-concept subset rather than all 271 concepts. Note. Standardization level (terminology-level versus information-model-level;), automation level (manual, semi-automatic, automatic), and validation type are defined in the Methods. Representation form is reported separately from the standardization level. Abbreviations: ICD-11, International Classification of Diseases 11th Revision; OMOP CDM, Observational Medical Outcomes Partnership Common Data Model; OWL, Web Ontology Language; RDF, Resource Description Framework; RxNorm, standardized nomenclature for clinical drugs (U.S. National Library of Medicine); TCM, traditional Chinese medicine; UMLS, Unified Medical Language System.

### 3.5. Representability Across Clinical Object Types

Organizing the mapping and modeling outcomes by the type of clinical object showed that the extent to which international standards could represent each object varied systematically by object type, from direct anchoring for acupoints to near-absence for patterns (Table 4). Acupoints could be anchored to mainstream terminologies, supported by a dedicated international standard (the WHO Standard Acupuncture Point Locations [44]), although this rested on a single study. Symptoms and disorders mapped extensively but fit only partially: in one study, 53.3% of the TCM symptom–UMLS links resolved to semantic types other than Sign, Symptom, or Finding; in a second, 18.8% (75/399) of the evaluated MeSH symptom terms (category C23.888) were classified as disease, syndrome, or pathology rather than symptom, and 28% (61/218) of the ICD-11 Chapter 26 disorders were fully representable in SNOMED CT. Compound herbal medicines could not be represented natively; they required model extension or decomposition rather than direct mapping. Pattern concepts were almost entirely absent: only 1 of 260 ICD-11 Chapter 26 patterns had an equivalent concept in the SNOMED CT International edition. The evidence for each object type came from different studies, standards, and denominators and in several places from a single study; the resulting arrangement is therefore a qualitative ordering of representational fit, not a measured or directly comparable scale, and its interpretation as a representability gradient is developed in the Discussion.

### 3.6. Form of Outputs and Their Relation to the Target Standards

The ten studies differed in the form of their outputs and in how those outputs related to the international standards. Four produced standalone resources anchored to existing standards: the acupuncture knowledge graph of Li et al. [38], the integrated symptom ontology of Shu et al. [40], the symptom database of Wu et al. [34], and the symptom knowledge base of Ruan et al. [39]. Two extended the model of a standard: the Chinese clinical drug knowledge base that Wang L et al. [43] built on the RxNorm information model, and the KIOM terminology extension of Park et al. [37]. Two proposed additions that had not been adopted into the standard: the new SNOMED CT concepts of Byun et al. [35] and the OMOP CDM fields identified by Cha et al. [41]. The remaining 2 assessed or demonstrated existing standards without producing new content [36,42]. No study reported that its additions or resources had been incorporated into the released content of a target standard. Where the relationship was stated explicitly, the content remained external: Park et al. [37] noted that its terminology extension had not been externally certified or integrated, and the additions of Byun et al. [35] were proposals rather than adopted content.

## 4. Discussion

### 4.1. Principal Findings

This review examined how the concepts of traditional East Asian medicine have been mapped or modeled into international health informatics standards and with what methods, coverage, and outcomes. The ten studies published between 2017 and 2026 met the inclusion criteria. The included studies were concentrated in TCM and TKM and centered on China and the Republic of Korea, with no study addressing Kampo or originating from Japan.

Three findings organize the discussion that follows. First, the studies engaged the three functional categories of interoperability standards unevenly, concentrating on the representation of shared meaning in terminologies, classifications, and ontologies while leaving the exchange category untouched. Second, the degree to which the standards accommodated traditional medicine concepts varied with the type of clinical object, an observed ordering that ran from acupoints, which mapped readily, to pattern, which existing standards could barely represent; we interpret this ordering as a representability gradient in the section below. Third, in response to these gaps, most of the studies converged on a common strategy, supplementing the standards locally through standalone resources, extensions, and proposals that remained outside the standards themselves.

### 4.2. Uneven Coverage Across Functional Categories

Health information standards fall into distinct functional categories. Terminologies, classifications, and ontologies establish shared meaning for clinical concepts; common data models specify how that information is structured for storage and analysis; and exchange standards govern how data move between systems [8]. Across the ten included studies, mapping and modeling activity was concentrated in certain categories and absent from others. Most studies targeted terminologies, classifications, and ontologies, anchoring TEAM concepts to standards such as SNOMED CT, the UMLS, ICD-11, MeSH, and RxNorm (Figure 3). Work on common data models was sparse and confined to a single common data model, the OMOP CDM, which was addressed by two studies [37,41]. No included study addressed exchange or messaging standards, and none used HL7 FHIR or an equivalent messaging standard (Figure 3).

This distribution indicates that the work captured here has concentrated on representational groundwork rather than on the mechanisms that enable data to move between systems. Anchoring a TEAM concept to a terminology or classification establishes what the concept means and makes it computable, but it does not by itself allow that concept to be exchanged between systems in a clinically usable message. Exchange standards such as HL7 FHIR provide that capability [7,12]. Their absence from the included literature suggests that the interoperability demonstrated in these studies remained at the level of concept representation rather than realized data exchange. The single common data model engaged, the OMOP CDM, supports structured storage and observational analysis but is not itself an exchange standard [11].

This pattern contrasts with mainstream health information interoperability research, in which exchange and messaging standards are routinely engaged alongside terminologies and data models. In a systematic review of semantic interoperability in electronic health records, content and exchange standards such as openEHR, HL7 FHIR, and the HL7 Clinical Document Architecture were each among the standards used by the analyzed studies [45], and a dedicated review of FHIR-based interoperability documented an extensive body of such work [46]. Relative to that broader field, these TEAM studies occupied a narrower range of functional categories, concentrating on establishing shared meaning without yet extending to the standards that move meaning between systems. A plausible interpretation is that this narrower range reflects an early developmental stage: because TEAM concepts depart substantially from the biomedical entity model, establishing shared meaning may be a prerequisite that is addressed before data models and exchange standards. This interpretation is not one that the included studies tested directly.

### 4.3. A Representability Gradient Across TEAM Clinical Objects

As the object-level results showed (Table 4), representability varied along this gradient, from acupoints at one end to patterns at the other. Each object type was represented by only a few studies, in some cases a single one, and the underlying metrics rest on different denominators and definitions (Table 4); the ordering should therefore be read as a hypothesis-generating interpretation of how far existing standards can hold each object type, not as a quantitative ranking or a comparison of the studies against one another.

At one end, acupoints were well anchored, although the evidence here comes from a single study. Li et al. [38] constructed an acupuncture knowledge graph on the WHO Standard Acupuncture Point Locations and linked points to SNOMED CT, MeSH, and UBERON. A dedicated international standard for the object made anchoring to mainstream terminologies achievable, although Li et al. [38] noted that terminologies such as SNOMED CT include acupoints only with limited information.

Symptoms and disorders occupied an intermediate position, where mapping was extensive but the fit was only partial (Table 4). The shortfall was conceptual as much as numerical. Over half of the TCM symptom–UMLS links reported by Ruan et al. [39] resolved to semantic types other than Sign, Symptom, or Finding, which the authors attributed to the broader scope of “symptom” in TCM than in biomedicine. Shu et al. [40], evaluating the symptom terms held in MeSH, found the same boundary mismatch within the standard itself: a notable share of terms carried non-symptom semantic types such as disease, syndrome, or pathology, a mismatch located inside MeSH rather than arising only when TEAM terms were mapped to it. For disorders, Lundholm and Bonacina [36] found only a minority of the ICD-11 Chapter 26 disorders fully representable in SNOMED CT (Table 4).

At the other end were compositional and inferential objects that biomedical standards could not hold natively. Compound herbal medicines required extension or decomposition rather than direct mapping. Wang et al. [43] extended the RxNorm information model to represent Chinese patent (herbal) drugs, which RxNorm could not accommodate natively, and Cha et al. [41] found that herbal medicine formulas could not be fitted into existing OMOP CDM fields, requiring supplementary fields to capture details such as herb composition. Park et al. [37] decomposed multi-herb prescriptions into individual herb components before mapping them to RxNorm, whose model represents single pharmaceutical ingredients rather than the herb combinations from which TKM decoctions derive their effect. In TEAM, the herbs in a formula are selected to balance and modify one another, so its intended action arises from their interaction and not from any single component [47], and a representation built from independent ingredients captures the parts but not this relational design. Across these studies, the reported coverage shows what a purpose-built local extension could capture, not what an international standard supports natively. It therefore demonstrates feasibility in principle rather than an established standard representation for compound herbal medicines.

Pattern differentiation sat at the extreme. In the same comparison, Lundholm and Bonacina [36] found that almost none of the ICD-11 Chapter 26 patterns had an equivalent SNOMED CT concept (Table 4), and Byun et al. [35] could map traditional-medicine pattern concepts only by drawing on the SNOMED CT Traditional Medicine Community Content, which the International edition alone did not provide. The ICD-11 Chapter 26 defines a pattern as a relatively temporary clinical picture that reflects the patient’s systemic response and brings together specific and non-specific manifestations [2], a composite construct that does not correspond to a single biomedical concept. A pattern is defined less by any individual finding than by the relationships among findings, the way symptoms, signs, and constitutional features co-occur and qualify one another, which is what makes it resist representation as a discrete entity. This resistance also points to where a solution might lie. Because a pattern is defined by the relations among findings, it may be expressed more effectively through compositional means, such as SNOMED CT post-coordination or ontology axioms that state the constituent findings and their relations, rather than as a single discrete code.

This gradient is consistent with how far each object departs from the entity-based, disease-centered assumptions on which biomedical standards are built. The designers of the ICD-11 Chapter 26 chose the term “disorder” rather than “disease” because many TEAM entities are defined by clusters of signs and symptoms instead of a defined etiology, which places them conceptually closer to symptom and finding categories than to diseases [2,17]. Objects closer to the biomedical frame, such as acupoints, which already have a dedicated international standard, and in part symptoms, correspond to the locations and findings these standards were designed to hold, whereas compositional objects such as herbal formulas and inferential constructs such as patterns do not. This aligns with longstanding observations that TEAM operates on a diagnostic paradigm distinct from the reductionist assumptions of biomedicine [14]. We therefore interpret conceptual distance from the biomedical paradigm as a plausible organizing explanation for this ordering, relating both to how readily an object maps and to whether it can be folded into mainstream standards or instead requires a parallel traditional-medicine resource; this is an interpretation of the assembled studies rather than a relationship those studies tested directly. Pattern differentiation, then, is not an incidental omission but the point at which the two paradigms diverge most sharply, which is also why the studies that addressed it relied on dedicated traditional-medicine extensions instead of direct mapping.

### 4.4. A Convergent Response: Local Supplementation Without Upstreaming

The included studies converged on a common response to these representational gaps. As the form of their outputs showed, most studies placed their TEAM-specific content outside the released standards, adding it as standalone resources, model extensions, or proposals rather than within the standards themselves. The strategy was consistent despite the heterogeneity of the artifacts: each study addressed the gaps it encountered by building alongside the standard rather than within it, and none reported that its additions had been incorporated into a released standard.

Because these additions remained external, the same representational gaps were met repeatedly rather than resolved once within the standards. Park et al. [37] noted that their terminology extension had not been externally certified or integrated, and the additions of Byun et al. [35] were proposals rather than adopted content; in each case, the work that closed a gap for one project did not become available within the standard for others. The consequence is duplicated effort, in which each project rebuilds representations that another has already produced, with no route for them to accumulate in the shared standards.

The artifacts were also produced by varied methods and validated against non-comparable reference points, with no shared benchmark across the studies. Taken together with their external character, this heterogeneity points to a field still at an early and fragmented stage, in which TEAM content is repeatedly re-represented in local artifacts rather than consolidated within the international standards themselves.

### 4.5. Implications and a Research and Standardization Agenda

The most consistent implication of these findings concerns the relationship between local supplementation and the international standards. Several studies produced extensions or mappings that addressed real representational gaps but remained outside the standards they extended. Park et al. [37] and Byun et al. [35] proposed terminology and SNOMED CT extensions, and Cha et al. [41] identified specific OMOP CDM fields that TEAM data would require. A priority for future work would be to carry such proposals through the formal change processes of the relevant standards. For SNOMED CT, for example, candidate TEAM content can be submitted through the Content Request Service or maintained within a dedicated extension namespace so that recurring requirements are met within the international standards rather than re-created locally by each project [48].

The studies that engaged the objects least amenable to direct mapping, particularly patterns, relied on dedicated traditional medicine content, whether the ICD-11 Chapter 26 or the SNOMED CT Traditional Medicine Community Content. The continued development of this dedicated content is therefore central to representing the parts of TEAM that general biomedical concepts cannot hold. Commentary from within the field has similarly argued that the traditional medicine content of ICD-11 captures only part of clinical practice and will need to be supplemented and upgraded [3], and the governance structures now in place for the ICD-11 Chapter 26 provide a route through which such expansion can occur [17].

Two further gaps follow from the earlier sections. No included study addressed the exchange category, so encoding TEAM concepts in exchange and messaging standards such as HL7 FHIR remains an open area for work directed at operational data sharing rather than representation alone. A concrete step would be the development of a FHIR Implementation Guide for TEAM data, defining the profiles, extensions, and value sets required to exchange TEAM concepts between systems [13]. The outputs also varied in how openly they were shared and how well they were maintained, and some repositories are already unreachable. This unevenness points to a need for stable, openly accessible deposit, without which extensions and mappings cannot be reused or built upon cumulatively. The case for data sharing in this research area has been made on the same grounds of reproducibility and cumulative progress [6].

A further direction concerns the methods by which TEAM content is mapped. The included studies already varied in automation: most mappings were produced by hand, but four combined algorithmic candidate generation with expert review, one used a predominantly automatic pipeline [39], and one constructed an acupuncture knowledge graph linked to mainstream terminologies [38]. This points to a role for artificial intelligence, including large language models, in future semantic mapping [4,5]. It could propose candidate correspondences at scale, surface the compositional and relational structure of constructs such as herbal formulas and patterns that resist direct one-to-one mapping, and extend coverage across the multi-lingual literature that a single-language search cannot reach. Such approaches would not remove the need for expert adjudication or for submission through the formal change processes of the standards. Their outputs would still require validation, which reinforces the need for shared validation benchmarks, such as agreed reference mapping sets and common evaluation metrics, that the heterogeneous validation practices observed here currently lack. Used in this way, artificial intelligence would accelerate the production of candidate representations rather than replace the human and governance work required to incorporate them.

Finally, the evidence base is uneven across the three traditions within this review’s scope. The TEAM frame follows the population definition of ICD-11 Chapter 26, which spans the practices of China, Japan, and the Republic of Korea [2]; the included studies, however, addressed only TCM and TKM, with no work on Kampo and none originating from Japan. This imbalance is better read as a finding about the field than as a reason to narrow the frame: standardization work directed at mainstream international standards has so far come almost entirely from Chinese and Korean settings, leaving Kampo recognized within ICD-11 Chapter 26 yet absent from the international standardization literature captured here. Kampo work was reached by the search but did not enter the included set: two Kampo studies were retrieved through the main database search and excluded, one as an ineligible publication type and one for making no mapping or modeling attempt, under the same criteria applied to all traditions. A concrete priority is therefore to develop mapping and modeling studies for Kampo, whose pattern and formula concepts are already recognized within ICD-11 Chapter 26 but have not yet been represented in the wider standards, in collaboration with Japanese research communities and national databases; extending such work to under-represented traditions would give a fuller account of how TEAM can be represented in international standards.

Taken together, these directions form a research and standardization agenda in which each priority addresses a specific gap identified here rather than a general aspiration. They are also interdependent: establishing shared meaning, structuring it in data models, and carrying it into exchange standards are stages of a single path toward operational interoperability, and each depends on the content produced at the earlier stages being consolidated within the international standards rather than rebuilt locally by each project.

### 4.6. Strengths and Limitations

This review has several strengths. It provides a focused synthesis of how traditional East Asian medicine concepts have been mapped or modeled into international health informatics standards, an intersection that prior reviews have not examined in this specific form. It also organizes a dispersed body of work into a structured account of where existing standards accommodate TEAM clinical objects, where representation breaks down, and how the field has compensated. Methodologically, it followed an established scoping review approach [22] and was reported in accordance with PRISMA-ScR [23], with a registered protocol on the OSF. The search covered four bibliographic databases, including IEEE Xplore to capture medical informatics conference proceedings, and was supplemented by citation searching and a structured gray literature search. Inter-rater reliability for title and abstract screening was assessed with several complementary agreement statistics rather than a single coefficient, an approach suited to the very low proportion of included records, under which chance-corrected measures such as Cohen’s kappa become unstable [27,28,29]. Full-text screening was conducted independently by two reviewers, who reached agreement on all eligibility decisions.

It also has limitations. The protocol was registered after the database searches, deduplication, and first-pass title and abstract screening had been completed, so the registration was in part retrospective; this is disclosed here and in the Methods. First-pass title and abstract screening was performed by a single reviewer, which departs from the dual-reviewer model recommended by JBI, although PRISMA-ScR recognizes a single reviewer with a second verifier as an acceptable alternative [22,23]. This deviation was mitigated in several ways: ambiguous records were retained as uncertain and carried forward to full-text screening rather than excluded at the title and abstract stage, inter-rater reliability was verified on a random 10% sample of screened records, and the full-text screening was conducted in duplicate. Because included records were almost absent from the reliability sample, the verification has limited power to detect a low rate of missed eligible studies among the roughly 90% of records that were not re-screened; the high agreement therefore reflects consistency on the sampled records rather than assurance that no eligible studies were missed elsewhere. The evidence base was also small and uneven: only 10 studies met the criteria, and several object types were represented by a single study, so the cross-object comparison is exploratory rather than confirmatory.

The search was also bounded. It was limited to reports written in English, which excludes the substantial traditional-medicine informatics literature published in Chinese, Korean, and Japanese and indexed in national databases such as CNKI (China), RISS and KMbase (Republic of Korea), and CiNii (Japan). Much TEAM work is conducted and published within these national research communities. The included literature characterized here is therefore best understood as the subset of standardization activity that has entered the international, English-language peer-reviewed literature, rather than the full global output. This visibility gap echoes a finding of the review itself, that TEAM standardization work tends to remain within local and national settings instead of being consolidated into the international standards. The included studies should therefore be read as a bounded, internationally visible sample, not a complete census, and the absences noted here, such as the lack of work on Kampo or on exchange-category standards, are observations about this sample rather than firm evidence that no such work exists. In particular, our eligibility criterion required the representation of TEAM concepts to be a study’s primary contribution, which may have excluded implementation or systems-integration work that used an exchange standard such as HL7 FHIR while addressing TEAM terminology only as one component.

A further bound concerned the search dates: the lower limit was not tested at the search stage. A confirmatory check performed during revision, in which the PubMed strategy was re-run without the lower date limit, identified no eligible study published before 2016, which indicates that the date window is unlikely to have excluded relevant work in that database. Because this check was confined to a single database, we cannot fully exclude the possibility of earlier eligible studies indexed elsewhere, although studies representing TEAM concepts in international standards appear predominantly in the informatics literature that PubMed indexes.

## 5. Conclusions

This review mapped how traditional East Asian medicine concepts have been represented in international health informatics standards, and within the internationally visible literature it identified a body of work still centered on establishing shared meaning. This work has clustered in terminologies and classifications, engaged the OMOP Common Data Model only occasionally, and did not extend to exchange standards such as HL7 FHIR. What a standard could represent depended on the type of clinical object at issue: objects close to the biomedical frame, such as acupuncture points, anchored readily to existing terminologies, whereas pattern differentiation, which sits furthest from that frame, remained largely out of reach. In response to these gaps, most studies built alongside the standards rather than within them, adding standalone resources, model extensions, or proposals that were never incorporated into a released standard. Because this content stays external, the same gaps are repeated by each new project instead of being resolved once. Progress is therefore likely to depend less on further local mapping than on carrying TEAM content through the formal change processes of the international standards. Alongside this, the field needs to develop dedicated content for the constructs that general biomedical terminologies cannot hold and to encode TEAM concepts in exchange standards so that representation can support operational data sharing.

## Figures and Tables

**Table 4:** Representation of TEAM clinical objects in mainstream international standards, arranged as a qualitative ordering of representational fit from acupoints to pattern.

Clinical Object	Mode of Representation	Supporting Evidence
Acupoint	Direct anchoring: a dedicated international standard exists for the object	Li 2026 [38]: anchored to the WHO Standard Acupuncture Point Locations; linked to SNOMED CT, MeSH, and UBERON
Symptom	Partial fit: concepts map, but a substantial share carries non-symptom semantic types	Ruan 2017 [39]: 92.0% of symptom–UMLS links correct, but 53.3% resolve to non-symptom types; Shu 2024 [40] ^a^: 18.8% of MeSH symptom-category terms classed as disease, syndrome, or pathology; Wu 2019 [34]: 1717 TCM symptoms curated to 961
Disorder ^b^	Partial fit: a minority fully equivalent in mainstream standards	Lundholm 2023 [36]: 28% (61/218) of ICD-11 Chapter 26 disorders fully representable in SNOMED CT International
Compound herbal medicine	Not held natively: requires model extension or decomposition	Wang L 2018 [43]: 90% coverage only after RxNorm model extension; Cha 2024 [41]: no OMOP CDM field for herbal-formula detail; Park 2026 [37]: 35% native, 100% after a KIOM extension
Pattern ^b^	Near-absent: almost no equivalent concept; requires dedicated traditional-medicine content	Lundholm 2023 [36]: 1/260 ICD-11 Chapter 26 patterns had a SNOMED CT equivalent; Byun 2024 [35] ^c^: 8/18 patterns mapped, only via the SNOMED CT Traditional Medicine Community Content

^a^ Shu 2024 [40]; the figure is from the study’s manual evaluation of the MeSH symptom category (C23.888): of 399 terms, 75 (18.8%) were classified as non-symptom types (32 disease, 22 syndrome, 21 pathology or physiology). ^b^ In the ICD-11 traditional medicine chapter, a disorder is a relatively constant clinical picture reflecting a main, constant pathologic process, whereas a pattern is a relatively temporary clinical picture reflecting the patient’s overall systemic response, recognized from the summarized whole picture observed through traditional-medicine theory [2]. ^c^ Byun 2024 [35]; values are pattern-specific (18 concepts), mapped via the SNOMED CT Traditional Medicine Community Content. Note. Rows are ordered along a qualitative gradient of representational fit rather than a numerical scale; the reported figures are heterogeneous and not directly comparable across rows (different denominators and definitions). Values shown after extension (Wang L 2018 [43], Park 2026 [37]) indicate the supplementation required, not native fit.

## Data Availability

No new data were created or analyzed in this study. Data sharing is not applicable to this article.

## Supplementary Materials

The following supporting information can be downloaded at: https://www.mdpi.com/article/10.3390/healthcare14183011/s1. File S1: PRISMA-ScR checklist; File S2: Full database search strategies; File S3: Screening and selection details, containing Table S1 (inter-rater reliability contingency table and agreement statistics) and Table S3 (reports excluded at full-text assessment, with reasons); File S4: Data charting spreadsheet, containing Table S2 (data charting form), Table S4 (complete charting record: mapping methodology, validation, tools, and quantitative outcomes), and Table S5 (auxiliary ontologies and vocabularies used in construction).

## Abbreviations

AC1	Agreement Coefficient 1 (Gwet’s)
AcuKG	Acupuncture Knowledge Graph
CDM	common data model
CM	conventional medicine
EHR	electronic health record
EMR	electronic medical record
HL7 FHIR	Health Level Seven Fast Healthcare Interoperability Resources
ICD-11	International Classification of Diseases, Eleventh Revision
ISPO	Integrated Symptom Phenotype Ontology
JBI	Joanna Briggs Institute
KM	Korean medicine
KIOM	Korea Institute of Oriental Medicine
LOINC	Logical Observation Identifiers Names and Codes
MeSH	Medical Subject Headings
NCCD	Normalized Chinese Clinical Drug knowledge base
OHDSI	Observational Health Data Sciences and Informatics
OMOP CDM	Observational Medical Outcomes Partnership Common Data Model
OSF	Open Science Framework
OWL	Web Ontology Language
PABAK	prevalence-adjusted bias-adjusted kappa
PCC	Population, Concept, and Context
PRISMA-ScR	Preferred Reporting Items for Systematic Reviews and Meta-Analyses extension for Scoping Reviews
RDF	Resource Description Framework
RxNorm	standardized nomenclature for clinical drugs (U.S. National Library of Medicine)
SNOMED CT	Systematized Nomenclature of Medicine Clinical Terms
SymMap	Symptom Mapping database
TCM	traditional Chinese medicine
TEAM	traditional East Asian medicine
TKM	traditional Korean medicine
TM	traditional medicine
TM1	Traditional Medicine Module I
UMLS	Unified Medical Language System
