# Supplementary material for: Representing Traditional East Asian Medicine in International Health Informatics Standards: A Scoping Review
Source: Healthcare (Basel). 2026 Sep 14;14(18):3011. doi: 10.3390/healthcare14183011 (PMC13606419; doi:10.3390/healthcare14183011)
Supplement: Supplementary file 1 [file healthcare-14-03011-s001.zip › S2. Full database search strategies.pdf]

Full database search strategies

This appendix presents the complete search strategy for each bibliographic database, with field tags, the limits applied, the date the search was executed, and the number of records retrieved, so that the searches can be reproduced. The strategy combined two concept blocks with the Boolean operator AND: Block 1, terms for traditional East Asian medicine (TEAM); and Block 2, terms for health informatics standardization and interoperability. Standards not named in the search (for example, UMLS, RxNorm, LOINC, and MeSH) were captured through the general concept terms. The coverage window was 1 January 2016 to 31 March 2026.

Database	Date searched	Limits applied	Records
PubMed	12 Apr 2026	Date range 2016/01/01–2026/03/31 and English language, both applied via PubMed filters	3,115
Embase	12 Apr 2026	Publication years 2016–2026 and entry-date range, and English language, applied within the query	9,308
Scopus	15 Apr 2026	PUBYEAR 2016–2026 (year level) and English language, applied within the query	3,955
IEEE Xplore	12 Apr 2026	Basic search, field set to All; publication year 2016–2026 applied via the year filter; language not restricted at search (English verified at screening)	385
Cochrane Library	12 Apr 2026	Considered but not used as a source; the 223 records retrieved were trial records duplicated from the other databases, with no native records of an eligible type	223
**Total (databases used)**			**16,763**

Note. Records shown are before de-duplication. The Cochrane Library records are not included in the total. The full query for each database is reproduced verbatim below.

PubMed (searched 12 April 2026; 3,115 records)

Block 1 (TEAM):

```
("Medicine, Traditional"[Mesh] OR "Medicine, Chinese Traditional"[Mesh] OR "Medicine, Korean Traditional"[Mesh] OR "Medicine, Kampo"[Mesh] OR "traditional medicine"[tiab] OR "traditional chinese medicine"[tiab] OR "traditional korean medicine"[tiab] OR "kampo"[tiab] OR "TCM"[tiab] OR "TKM"[tiab] OR "Traditional East Asian Medicine"[tiab] OR "East Asian Traditional Medicine"[tiab] OR "EATM"[tiab])
```

AND Block 2 (health informatics standardization and interoperability):

```
("Systematized Nomenclature of Medicine"[Mesh] OR "Health Level Seven"[Mesh] OR "Medical Informatics"[Mesh] OR "Terminology as Topic"[Mesh] OR "Vocabulary, Controlled"[Mesh] OR
```

"Biological Ontologies"[Mesh] OR "Standardization"[Mesh] OR "SNOMED"[tiab] OR "SNOMED CT"[tiab] OR "ICD-11"[tiab] OR "ICD 11"[tiab] OR "ICD-10"[tiab] OR "ICD 10"[tiab] OR "ICTM"[tiab] OR "International Classification of Traditional Medicine"[tiab] OR "OMOP"[tiab] OR "CDM"[tiab] OR "common data model"[tiab] OR "data model"[tiab] OR "HL7"[tiab] OR "FHIR"[tiab] OR "ISO standard"[tiab] OR "ISO standards"[tiab] OR "International Organization for Standardization"[tiab] OR "ontology"[tiab] OR "ontologies"[tiab] OR "terminology"[tiab] OR "terminologies"[tiab] OR "controlled vocabulary"[tiab] OR "interoperability"[tiab] OR "standardization"[tiab] OR "standardisation"[tiab] OR "mapping"[tiab] OR "medical informatics"[tiab] OR "health informatics"[tiab])

Embase — Embase.com (searched 12 April 2026; 9,308 records)

('traditional medicine'/exp OR 'chinese medicine'/exp OR 'korean medicine'/exp OR 'kampo'/exp OR 'traditional medicine':ti,ab OR 'traditional chinese medicine':ti,ab OR 'traditional korean medicine':ti,ab OR 'kampo':ti,ab OR 'tcm':ti,ab OR 'tkm':ti,ab OR 'traditional east asian medicine':ti,ab OR 'east asian traditional medicine':ti,ab OR 'eatm':ti,ab) AND ('systematized nomenclature of medicine'/exp OR 'health level seven'/exp OR 'medical informatics'/exp OR 'terminology'/exp OR 'controlled vocabulary'/exp OR 'ontology'/exp OR 'standardization'/exp OR 'snomed':ti,ab OR 'snomed ct':ti,ab OR 'icd-11':ti,ab OR 'icd 11':ti,ab OR 'icd-10':ti,ab OR 'icd 10':ti,ab OR 'ictm':ti,ab OR 'international classification of traditional medicine':ti,ab OR 'omop':ti,ab OR 'cdm':ti,ab OR 'common data model':ti,ab OR 'data model':ti,ab OR 'hl7':ti,ab OR 'fhir':ti,ab OR 'iso standard':ti,ab OR 'iso standards':ti,ab OR 'international organization for standardization':ti,ab OR 'ontology':ti,ab OR 'ontologies':ti,ab OR 'terminology':ti,ab OR 'terminologies':ti,ab OR 'controlled vocabulary':ti,ab OR 'interoperability':ti,ab OR 'standardization':ti,ab OR 'standardisation':ti,ab OR 'mapping':ti,ab OR 'medical informatics':ti,ab OR 'health informatics':ti,ab) AND [2016-2026]/py AND [english]/lim AND [01-01-2016]/sd NOT [01-04-2026]/sd

Scopus (searched 15 April 2026; 3,955 records)

TITLE-ABS-KEY (("Medicine, Traditional" OR "Medicine, Chinese Traditional" OR "Medicine, Korean Traditional" OR "Medicine, Kampo" OR "traditional medicine" OR "traditional chinese medicine" OR "traditional korean medicine" OR "kampo" OR "TCM" OR "TKM" OR "Traditional East Asian Medicine" OR "East Asian Traditional Medicine" OR "EATM") AND ("Systematized Nomenclature of Medicine" OR "Health Level Seven" OR "Medical Informatics" OR "Terminology as Topic" OR "Vocabulary, Controlled" OR "Biological Ontologies" OR "medical informatics" OR "health informatics" OR "SNOMED" OR "SNOMED CT" OR "ICD-11" OR "ICD 11" OR "ICD-10" OR "ICD 10" OR "ICTM" OR "International Classification of Traditional Medicine" OR "OMOP" OR "CDM" OR "common data model" OR "data model" OR "HL7" OR "FHIR" OR "ISO standard" OR "ISO standards" OR "International Organization for Standardization" OR "ontology" OR "ontologies" OR "terminology" OR "terminologies" OR "controlled vocabulary" OR "interoperability" OR "data standardization" OR "terminology standardization" OR "data mapping" OR "concept mapping")) AND PUBYEAR > 2015 AND PUBYEAR < 2027 AND (LIMIT-TO (LANGUAGE , "English"))

IEEE Xplore (searched 12 April 2026; 385 records)

Basic search with the search field set to All; publication year limited to 2016–2026 via the year filter.

```
("traditional medicine" OR "traditional chinese medicine" OR "traditional korean medicine" OR "kampo" OR "TCM" OR "TKM" OR "Traditional East Asian Medicine" OR "East Asian Traditional Medicine" OR "EATM") AND ("SNOMED" OR "SNOMED CT" OR "ICD-11" OR "ICD 11" OR "ICD-10" OR "ICD 10" OR "ICTM" OR "International Classification of Traditional Medicine" OR "OMOP" OR "CDM" OR "common data model" OR "data model" OR "HL7" OR "FHIR" OR "ISO standard" OR "ISO standards" OR "International Organization for Standardization" OR "ontology" OR "ontologies" OR "terminology" OR "terminologies" OR "controlled vocabulary" OR "interoperability" OR "standardization" OR "standardisation" OR "mapping" OR "medical informatics" OR "health informatics")
```

Cochrane Library (searched 12 April 2026; 223 records; considered but not used)

Retrieved records were trial records duplicated from the other databases; the database returned no native records of an eligible type and was therefore not used as a source.

Block 1 (TEAM):

```
[mh "Medicine, Traditional"] OR [mh "Medicine, Chinese Traditional"] OR [mh "Medicine, Korean Traditional"] OR [mh "Medicine, Kampo"] OR "traditional medicine":ti,ab,kw OR "traditional chinese medicine":ti,ab,kw OR "traditional korean medicine":ti,ab,kw OR "kampo":ti,ab,kw OR "TCM":ti,ab,kw OR "TKM":ti,ab,kw OR "Traditional East Asian Medicine":ti,ab,kw OR "East Asian Traditional Medicine":ti,ab,kw OR "EATM":ti,ab,kw
```

AND Block 2:

```
[mh "Systematized Nomenclature of Medicine"] OR [mh "Health Level Seven"] OR [mh "Medical Informatics"] OR [mh "Terminology as Topic"] OR [mh "Vocabulary, Controlled"] OR [mh "Biological Ontologies"] OR [mh "Standardization"] OR "medical informatics":ti,ab,kw OR "health informatics":ti,ab,kw OR "SNOMED":ti,ab,kw OR "SNOMED CT":ti,ab,kw OR "ICD-11":ti,ab,kw OR "ICD 11":ti,ab,kw OR "ICD-10":ti,ab,kw OR "ICD 10":ti,ab,kw OR "ICTM":ti,ab,kw OR "International Classification of Traditional Medicine":ti,ab,kw OR "OMOP":ti,ab,kw OR "CDM":ti,ab,kw OR "common data model":ti,ab,kw OR "data model":ti,ab,kw OR "HL7":ti,ab,kw OR "FHIR":ti,ab,kw OR "ISO standard":ti,ab,kw OR "ISO standards":ti,ab,kw OR "International Organization for Standardization":ti,ab,kw OR "ontology":ti,ab,kw OR "ontologies":ti,ab,kw OR "terminology":ti,ab,kw OR "terminologies":ti,ab,kw OR "controlled vocabulary":ti,ab,kw OR "interoperability":ti,ab,kw OR "standardization":ti,ab,kw OR "standardisation":ti,ab,kw OR "mapping":ti,ab,kw
```
