# Supplementary material for: Representing Traditional East Asian Medicine in International Health Informatics Standards: A Scoping Review
Source: Healthcare (Basel). 2026 Sep 14;14(18):3011. doi: 10.3390/healthcare14183011 (PMC13606419; doi:10.3390/healthcare14183011)
Supplement: Supplementary file 1 [file healthcare-14-03011-s001.zip › S3. Screening and selection details.pdf]

Screening and selection details

This appendix supports the study-selection process described in the Methods and summarized in the PRISMA-ScR flow diagram (Figure 1). *Table S1* reports the inter-rater reliability verification of title and abstract screening. *Table S3* lists every report excluded at full-text assessment, with the primary reason for each. R1 denotes the primary reviewer and R2 the senior (verifying) reviewer.

Table S1. Inter-rater reliability of title and abstract screening on a 10% random verification sample (n=1090).

	R2: Include	R2: Maybe	R2: Exclude	Row total
**R1: Include**	2	1	0	3
**R1: Maybe**	0	0	0	0
**R1: Exclude**	0	0	1087	1087
**Column total**	**2**	**1**	**1087**	**1090**

Measure	Value
Verification sample	1090 records (10% random sample of 10,901 screened records)
Included-record prevalence in sample	3/1090 (0.28%)
Records in agreement	1089 of 1090
Raw percent agreement	99.91%
Cohen κ	0.833 (95% CI not estimable under extreme prevalence imbalance)
PABAK (prevalence-adjusted bias-adjusted kappa)	0.998
Gwet AC1	0.999

Note. The single disagreement concerned one record marked Include by R1 and Maybe by R2; both categories were carried forward to full-text assessment, so no eligible record was excluded at this stage. Percent agreement, Cohen κ, PABAK, and Gwet AC1 were computed from the contingency table above. Because included records were very rare (0.28%), Cohen κ is downwardly biased under the skewed marginal distribution, and the prevalence-adjusted measures (PABAK, Gwet AC1) are reported alongside it; the assessment of agreement rests primarily on the raw percent agreement and on the number of false-negative exclusions recovered.

Table S3. Reports excluded at full-text assessment, with the primary reason for exclusion (n=18: 14 from database searching, 4 from citation searching).

#	Study	Title	Search source	Primary reason for exclusion
1	Liang Z (2019)	An XHTML solution for a secure EDC system for traditional Chinese medicine clinics based on ICD-11 MMS database and e-signature	Database	No mapping or application attempt
2	Nie Y (2023)	Construction and application of traditional Chinese medicine clinical terminological system based on ISO 19465:2017	Database	No mapping or application attempt
3	Tong L (2024)	Research on knowledge framework and classification of traditional Chinese medicine diagnosis and treatment for myopia	Database	No mapping or application attempt
4	Reddy B (2022)	Incorporation of complementary and traditional medicine in ICD-11	Database	Ineligible publication type
5	Tian Y (2022)	Research and implementation of real world traditional Chinese medicine clinical scientific research information electronic medical record sharing system	Database	No mapping or application attempt
6	Wang W (2025)	Applied LLM for the construction of traditional Chinese medicine tongue diagnosis ontology	Database	No mapping or application attempt
7	Watanabe K (2019)	Relationship between Kampo pattern diagnoses and ICD classification	Database	Ineligible publication type
8	Wu X (2021)	Relationship between conventional medicine chapters in ICD-10 and Kampo pattern diagnosis: a cross-sectional study	Database	No mapping or application attempt
9	Long H (2017)	Outline of the construction and application of a GFO-based TCM diagnoses ontology for syndrome differentiation of psoriasis vulgaris	Database	Foundational or upper-ontology alignment
10	Shi F (2024)	A semantic ontology of acupoints in traditional Chinese medicine aligning with the Basic Formal Ontology	Database	Foundational or upper-ontology alignment
11	Long H (2019)	An ontological framework for the formalization, organization and usage of TCM-Knowledge	Database	Foundational or upper-ontology alignment
12	Selvaraj S (2020)	TKM ontology integration and visualization	Database	No mapping or application attempt
13	Yu T (2015)	Towards a TCM domain ontology: standardization, ontology engineering, and applications	Database	Outside the time frame
14	Zhou H	Complementary and integrative health	Database	Insufficient TEAM-

#	Study	Title	Search source	Primary reason for exclusion
	(2024)	information in the literature: its lexicon and named entity recognition		specific analysis
15	Guan H & Xie D (2017)	Research on building of acupuncture domain ontology	Citation (backward; from Li Y 2026)	No mapping or application attempt
16	Shojaee-Mend H et al (2019)	Development and evaluation of ontologies in traditional medicine: a review study	Citation (backward; from Lundholm 2023)	Ineligible publication type
17	Han X (2021)	Acupuncture and Tuina knowledge graph for ancient literature of traditional Chinese medicine	Citation (backward; from Li Y 2026)	No mapping or application attempt
18	Li X (2024)	Acupuncture and tuina knowledge graph with prompt learning	Citation (backward; from Li Y 2026)	No mapping or application attempt

Note. Reason categories correspond to the eligibility criteria: “no mapping or application attempt” and “insufficient TEAM-specific analysis” to exclusion criterion 2 and 5; “foundational or upper-ontology alignment” to criterion 3; “ineligible publication type” to criterion 4; “outside the time frame” to the inclusion window (January 2016 to March 2026). Counts by reason match the PRISMA-ScR flow diagram (Figure 1): database branch, no mapping or application attempt (7), foundational or upper-ontology alignment (3), ineligible publication type (2), outside the time frame (1), insufficient TEAM-specific analysis (1); citation branch, no mapping or application attempt (3), ineligible publication type (1). TEAM, traditional East Asian medicine.

References

The reference numbers below correspond to the # column in Table S3.

1. Liang Z, Liu B, Kong J, Lu Y, Xu N. An XHTML solution for a secure EDC system for traditional Chinese medicine clinics based on ICD-11 MMS database and e-signature. In: 2019 IEEE International Conference on Bioinformatics and Biomedicine (BIBM). IEEE; 2019:2634. doi:10.1109/BIBM47256.2019.8983155

2. Nie Y, Jia L, Gao B, Cui M, Gao Y, Li H. Construction and application of traditional Chinese medicine clinical terminological system based on ISO 19465:2017. Guidelines and Standards of Chinese Medicine. 2023. doi:10.1097/gscm.0000000000000003

3. Tong L, Cui Y, Chen G, Liu S, Zhang Y, Liu B. Research on knowledge framework and classification of traditional Chinese medicine diagnosis and treatment for myopia. In: 2024 IEEE International Conference on Bioinformatics and Biomedicine (BIBM). IEEE; 2024. doi:10.1109/BIBM62325.2024.10821787

4. Reddy B, Fan AY. Incorporation of complementary and traditional medicine in ICD-11. BMC Med Inform Decis Mak. 2022;21(Suppl 6):381. doi:10.1186/s12911-022-01913-7

5. Tian Y, Xie Q, Zhang R, Zhou X, Wen T, Zhang L. Research and implementation of real world traditional Chinese medicine clinical scientific research information electronic medical record sharing system. In: 2022 IEEE International Conference on Bioinformatics and Biomedicine (BIBM). IEEE; 2022. doi:10.1109/BIBM55620.2022.9994972

6. Wang W, Yao K, Ge P, Lu Y, Wang Z, Zhu Y. Applied LLM for the construction of traditional Chinese medicine tongue diagnosis ontology. In: 2025 IEEE International Conference on Bioinformatics and Biomedicine (BIBM). IEEE; 2025. doi:10.1109/BIBM66473.2025.11356313

7. Watanabe K, Maeda A, Yoshino T, Nakamura T. Relationship between Kampo pattern diagnoses and ICD classification [conference abstract P012]. Adv Integr Med. 2019. doi:10.1016/j.aimed.2019.03.167

8. Wu X, Le TK, Maeda-Minami A, Yoshino T, Horiba Y, Mimura M, Watanabe K. Relationship between conventional medicine chapters in ICD-10 and Kampo pattern diagnosis: a cross-sectional study. Front Pharmacol. 2021;12:751403. doi:10.3389/fphar.2021.751403

9. Long H, Zhu Y, Jia L, Wang J, Herre H. Outline of the construction and application of a GFO-based TCM diagnoses ontology for syndrome differentiation of psoriasis vulgaris. In: 2017 IEEE 19th International Conference on e-Health Networking, Applications and Services (Healthcom). IEEE; 2017. doi:10.1109/HealthCom.2017.8210829

10. Shi F, Zhu T, Ouyang B, Ning H. A semantic ontology of acupoints in traditional Chinese medicine aligning with the Basic Formal Ontology. In: 2024 International Conference on Identification, Information and Knowledge in the Internet of Things (IIKI). IEEE; 2024. doi:10.1109/IIKI65561.2024.00039

11. Long H, Zhu Y, Jia L, Gao B, Liu J, Liu L, Herre H. An ontological framework for the formalization, organization and usage of TCM-Knowledge. BMC Med Inform Decis Mak. 2019;19. doi:10.1186/s12911-019-0760-9

12. Selvaraj S, Choi E. TKM ontology integration and visualization. In: Proceedings of the 3rd International Conference on Software Engineering and Information Management (ICSIM). ACM; 2020. doi:10.1145/3378936.3378976

13. Yu T, Cui M, Jia L. Towards a TCM domain ontology: standardization, ontology engineering, and applications. In: 2015 8th International Conference on Biomedical Engineering and Informatics (BMEI). IEEE; 2015. doi:10.1109/BMEI.2015.7401556

14. Zhou H, Austin R, Lu SC, Silverman GM, Zhou Y, Kilicoglu H, Xu H, Zhang R. Complementary and integrative health information in the literature: its lexicon and named entity recognition. J Am Med Inform Assoc. 2024;31(2):426. doi:10.1093/jamia/ocad216

15. Guan H, Xie D. Research on building of acupuncture domain ontology. In: 2017 IEEE 19th International Conference on e-Health Networking, Applications and Services (Healthcom). IEEE; 2017. doi: 10.1109/HealthCom.2017.8210821

16. Shojaee-Mend H, Ayatollahi H, Abdolahadi A. Development and evaluation of ontologies in traditional medicine: a review study. Methods Inf Med. 2019;58(6):194-204. doi:10.1055/s-0040-1702236

17. Han X, Li X, Liang Y, Wang X, Xu D, Guan R. Acupuncture and Tuina knowledge graph for ancient literature of traditional Chinese medicine. In: 2021 IEEE International Conference on Bioinformatics and Biomedicine (BIBM). IEEE; 2021. doi: 10.1109/BIBM52615.2021.9669508

18. Li X, Han X, Wei S, Liang Y, Guan R. Acupuncture and tuina knowledge graph with prompt learning. Front Big Data. 2024;7:1346958. doi:10.3389/fdata.2024.1346958
